# Explicitness in SMILES representation via ExACT: improved tokenization for aqueous solubility prediction

**DOI:** 10.1186/s13321-026-01263-w

**Published:** 2026-08-04

**Authors:** Domagoj Begušić, Damir Pintar, Zlatko Smole, Ernest Meštrović

**Affiliations:** 1 University of Zagreb Faculty of Electrical Engineering and Computing, Zagreb, Croatia; 2 Modalis Ltd., Zagreb, Croatia; 3 University of Zagreb Faculty of Chemical Engineering and Technology, Zagreb, Croatia

**Keywords:** ExACT, Tokenization, Machine learning, Water solubility

## Abstract

The representation of molecular structure in textual form plays a central role in data-driven cheminformatics, not just for deep learning models that often rely on sequence-based inputs, but also for classic machine learning pipelines which are still relevant in this field. The Simplified Molecular Input Line Entry System (SMILES) representation and its explicit variants differ substantially, yet the implications of this difference for tokenization and downstream property prediction remain insufficiently characterized. This paper systematically investigates the role of explicitness in SMILES representation and its impact on aqueous solubility prediction. Four SMILES variations ranging from basic and canonical to explicit and explicit canonical are reviewed to illustrate how increasing explicit chemical detail alters the available information content. Building on this analysis, a novel tokenization approach termed ExACT (Explicit Atom level Context Tokenization) is introduced, which directly leverages atom level explicitness and local chemical context. In addition, a general strategy for enhancing existing tokenization methods through increased explicitness is proposed and demonstrated by upgrading the current state of the art atom-in-SMILES method to an explicit variant. All approaches are evaluated on the AqSolDB dataset using consistent machine learning pipelines and cross validation protocols. The results show that increased explicitness systematically improves predictive performance across tokenization strategies, yielding higher coefficients of determination, lower mean absolute and root mean squared errors, and reduced variance across folds. The proposed ExACT method achieves the best overall performance, largely outperforming traditional molecular fingerprint representations while remaining statistically comparable to the best competing tokenization methods. Furthermore, the efficiency analysis demonstrated that ExACT offers additional computational advantages, including lower dimensionality and the elimination of the decoding step, resulting in faster feature computation. These findings demonstrate that explicit SMILES representations encode chemically meaningful information that can be effectively exploited through context-aware tokenization, providing an interpretable and computationally efficient alternative representation for molecular property prediction.

**Scientific contribution**

This work investigates the role of explicitness in SMILES and its impact on aqueous solubility prediction, proposes a novel ExACT tokenization method and a general strategy for enhancing existing tokenization methods through increased explicitness.

**Supplementary Information:**

The online version contains supplementary material available at https://doi.org/10.1186/s13321-026-01263-w.

## Introduction and related work

Molecular representation underpins modern computational approaches in cheminformatics [1, 2]. Textual representations in particular serve as the basis for both classical machine learning pipelines and contemporary sequence-based models [3]. Among textual formats, the Simplified Molecular Input Line Entry System (SMILES) [4] has achieved near universal adoption due to its compactness and compatibility with standard cheminformatics toolkits. SMILES encodes molecular structure as a sequence of characters reflecting atoms, bonds, branches, and ring closures, thereby enabling straightforward storage, retrieval, and processing of chemical information. Despite its widespread use, SMILES suffers from inherent limitations related to representational ambiguity, token granularity, and insufficient encoding of local chemical context, all of which can influence downstream model performance [5].

Tokenization constitutes a critical preprocessing step in sequence based modeling. Although the term tokenization is most commonly associated with natural language processing and representation learning, it more generally denotes the process of transforming a sequential representation into a set of discrete symbolic units (tokens). In the present work, SMILES strings are decomposed into chemically meaningful units that serve as the fundamental elements of subsequent molecular representations. Within natural language processing, tokenization methods strongly affect model convergence, generalization, and the extraction of meaningful patterns from sequence inputs. Similarly, tokenization methods applied to SMILES significantly influence the information content available to models and the chemical interpretability of the learned features [6]. Conventional atom-wise or character-wise SMILES tokenization treats all occurrences of the same atomic symbol as identical tokens, thereby ignoring differences in chemical environment that are fundamental to molecular behavior. This approach, while straightforward, is limited by its lack of chemical context; atoms with identical atomic numbers but differing local environments are represented indistinguishably, which can obscure critical structural information during model training. Recent work has highlighted that such generic token representation can lead to token degeneration issues in language modeling tasks [7].

Research on SMILES tokenization methods has gained increasing attention with the rise of sequence-based machine learning models adapted from natural language processing [8]. Advanced tokenization strategies such as atom-in-SMILES (AIS) [9] enrich tokens with local chemical environment information derived from adjacent atom environments, demonstrating improved performance on translation and prediction tasks compared to traditional methods. Empirical evaluations show that AIS can outperform conventional tokenization approaches by generating more chemically informative token sequences and reducing repetitive token patterns that degrade model performance [7, 10].

Additional tokenization strategies further emphasize the importance of capturing substructure patterns and data-driven token vocabularies. SMILES Pair Encoding (SPE) [11] learns frequent SMILES substrings as tokens from large chemical datasets, enhancing representation by including human-readable subunits extending beyond atom-level tokens. This method has been shown to improve performance for both molecular generation and quantitative structure-activity relationship prediction tasks relative to atom-level tokenization alone. Recent work has also focused on atomically complete tokenizers designed to better cover the complexity of the SMILES language under the OpenSMILES specification and address limitations in vocabularies used by molecular foundation models [12]. Comparative studies of SMILES and alternative string representations such as SELFIES further underscore the influence of representation and tokenization choice on predictive performance and structural fidelity in learned models [13].

Beyond tokenization itself, variations in SMILES representation including canonical, explicit, and explicit canonical forms further shape the distribution of tokens. Canonical SMILES enforces a deterministic ordering of atoms but retains an abstracted form of chemical detail. Explicit SMILES incorporates additional information such as explicit hydrogen atoms and bond specifications, increasing the granularity of the representation and potentially exposing models to deeper structural features. Explicit canonical forms combine deterministic atom ordering with full explicit specification, yielding representations that maximize explicit chemical detail. While representational richness is hypothesized to facilitate more informative tokenization, systematic analysis of how increasing explicitness impacts tokenization quality and predictive performance has not been comprehensively addressed.

In parallel with tokenization research, multiple studies have evaluated the use of SMILES and derived representations for molecular property prediction. Early work demonstrated that recurrent neural network models trained directly on SMILES strings can learn predictive patterns for chemical properties such as solubility and toxicity without explicit feature engineering, establishing SMILES-based deep learning models as viable alternatives to descriptor-based methods [14–16]. More recent explorations have combined SMILES representations with deep neural network architectures to predict diverse properties [17], while hybrid models integrating both SMILES and vector-based fingerprints indicate potential performance gains through multimodal feature integration [3, 18–20]. Transformer-based architectures and attention mechanisms have further amplified the importance of tokenization design choices for downstream predictive performance [8, 21, 22].

Aqueous solubility is a fundamental physicochemical property that governs the applicability of chemical compounds across a broad range of domains, including pharmaceutical drug discovery and development, agrochemical design, and cosmetic formulation [23, 24]. In drug development, solubility directly influences bioavailability, formulation strategy, and clinical efficacy, making it one of the earliest and most critical filters in compound selection [25]. Beyond pharmaceuticals, solubility also plays a decisive role in determining the environmental fate and biological activity of agrochemicals, as well as the stability and performance of active ingredients in cosmetics and related industries [26]. Reliable computational prediction of aqueous solubility therefore represents a high-value target in cheminformatics, with implications spanning early-stage virtual screening to the rational design of compounds with tailored physicochemical profiles [27, 28].

Aqueous solubility prediction exemplifies a molecular property that is highly sensitive to structural subtleties including hydrogen bonding patterns and local chemical environments [29]. The development of benchmark datasets such as AqSolDB [28] which consolidates experimental solubility measurements with validated SMILES representations for thousands of compounds has enabled comparisons across descriptor-based, fingerprint-based, and sequence-based modeling approaches. Prior studies have used AqSolDB to train models incorporating SMILES randomization and descriptor augmentation to improve performance [30], while machine learning protocols have also been adapted to account for the inherent noise and variability in experimental solubility data [17, 31]. Benchmark efforts in related computational property prediction challenges further emphasize the difficulty of accurately modeling physical properties from structural representations, motivating continued innovation in both representation and algorithmic design [32].

Despite these advancements, systematic investigation of how increasing explicit chemical detail in SMILES representations influences token quality and predictive performance remains underdeveloped. This paper addresses this gap by systematically exploring the role of explicitness in SMILES representations and its implications for tokenization and aqueous solubility prediction. We first review established tokenization methods across four SMILES formats spanning basic to explicit canonical representations, illustrating how increasing explicit chemical detail alters the information available for tokenization. Building on this analysis, we introduce ExACT (Explicit Atom level Context Tokenization), a novel tokenization method that leverages explicit representation to capture atom level context more effectively. Additionally, we present a strategy for enhancing existing tokenization methods through explicitness, demonstrated by upgrading the current state of the art atom-in-SMILES approach to an explicit variant. Finally, we evaluate the performance of all methods using consistent machine learning pipelines on the AqSolDB dataset. Results indicate that increased explicitness systematically improves predictive performance, yielding models with higher coefficients of determination, lower mean absolute and root mean squared errors, and reduced variance across cross validation folds. These findings underscore the importance of explicit chemical detail for tokenization in cheminformatics and provide a structured pathway for advancing sequence based property prediction without reliance on graph based neural architectures.

## Methodology

This section details the overall machine learning process implemented in this study and describes the specific methods used to evaluate the impact of explicit SMILES tokenization on aqueous solubility prediction. First, the general workflow of data preprocessing, representation, model training, and evaluation is outlined. Next, five methodological components are presented in dedicated subsections: (I) process overview, (II) used data characteristics and preprocessing, (III) the proposed explicit SMILES tokenization method ExACT, (IV) the approach of integrating explicitness in AIS tokenization method, and (V) the selection and rationale for the machine learning models employed.

### Process overview

The machine learning workflow employed in this study is structured to ensure consistent processing across datasets and tokenization variants. Two datasets were used: AqSolDB [28] and its new highly curated version that we will refer to as AqSolDBc [33] which was primarily used to extract the adjusted aqueous solubility values. The used datasets are described in detail in the next subsection. The input representation is SMILES strings from the AqSolDB dataset.

Molecular structures were represented using SMILES strings obtained from AqSolDB. The target variables extracted from AqSolDB included aqueous solubility, molecular weight, lipophilicity, and molar refractivity. Also, adjusted aqueous solubility values from AqSolDBc were incorporated as an additional target variable. The analysis presented in this paper focuses on aqueous solubility and molecular weight, while the results for lipophilicity, molar refractivity and adjusted aqueous solubility are provided in the supplementary materials (Appendix C).

The pipeline begins with preprocessing of the raw SMILES strings. First, invalid and duplicate SMILES strings were excluded. Then, all necessary SMILES representation formats were calculated for each molecule. Next, tokenization methods are applied to each appropriate SMILES representation variant to decompose the SMILES sequences into discrete tokens appropriate for encoding. The resulting token sequences are transformed into numerical feature vectors using term frequency inverse document frequency vectorization (TF-IDF). In this representation, each token is assigned a weight proportional to its frequency within a given sequence and inversely proportional to its occurrence across the full dataset, thereby reducing the influence of prevalent tokens. Using a simple, model-agnostic vectorization method allows the evaluation to focus on the impact of the proposed tokenization and explicitness strategies, without introducing additional variability from learned representation methods.

To obtain robust and statistically reliable performance estimates, model evaluation was conducted using a repeated 5-fold cross validation procedure with five repetitions. For each repetition, the dataset was randomly partitioned into five folds, with four folds used for training and one fold reserved for testing. This process was repeated until each fold had served as the test set once, resulting in a total of 25 evaluation runs for each experimental configuration. The study employed XGBoost Regressor and CatBoost Regressor models using their default hyperparameter settings. Fixed hyperparameters were intentionally retained throughout all experiments to reduce computational complexity and to avoid potential performance inflation associated with extensive hyperparameter optimization. This design choice ensures that observed differences in predictive performance can be more directly attributed to the investigated tokenization methods rather than to model-specific tuning procedures.

Model performance was evaluated using the coefficient of determination ($R^2$), mean absolute error (*MAE*), and root mean squared error (*RMSE*). In addition to predictive performance, the total computational time required to execute the complete machine learning pipeline, from feature generation based on the tokenized SMILES representations to model training and evaluation, was recorded. The dimensionality of each generated feature space ($n_{features}$) was also tracked to quantify the complexity of the resulting molecular representations.

**Fig. 1:**
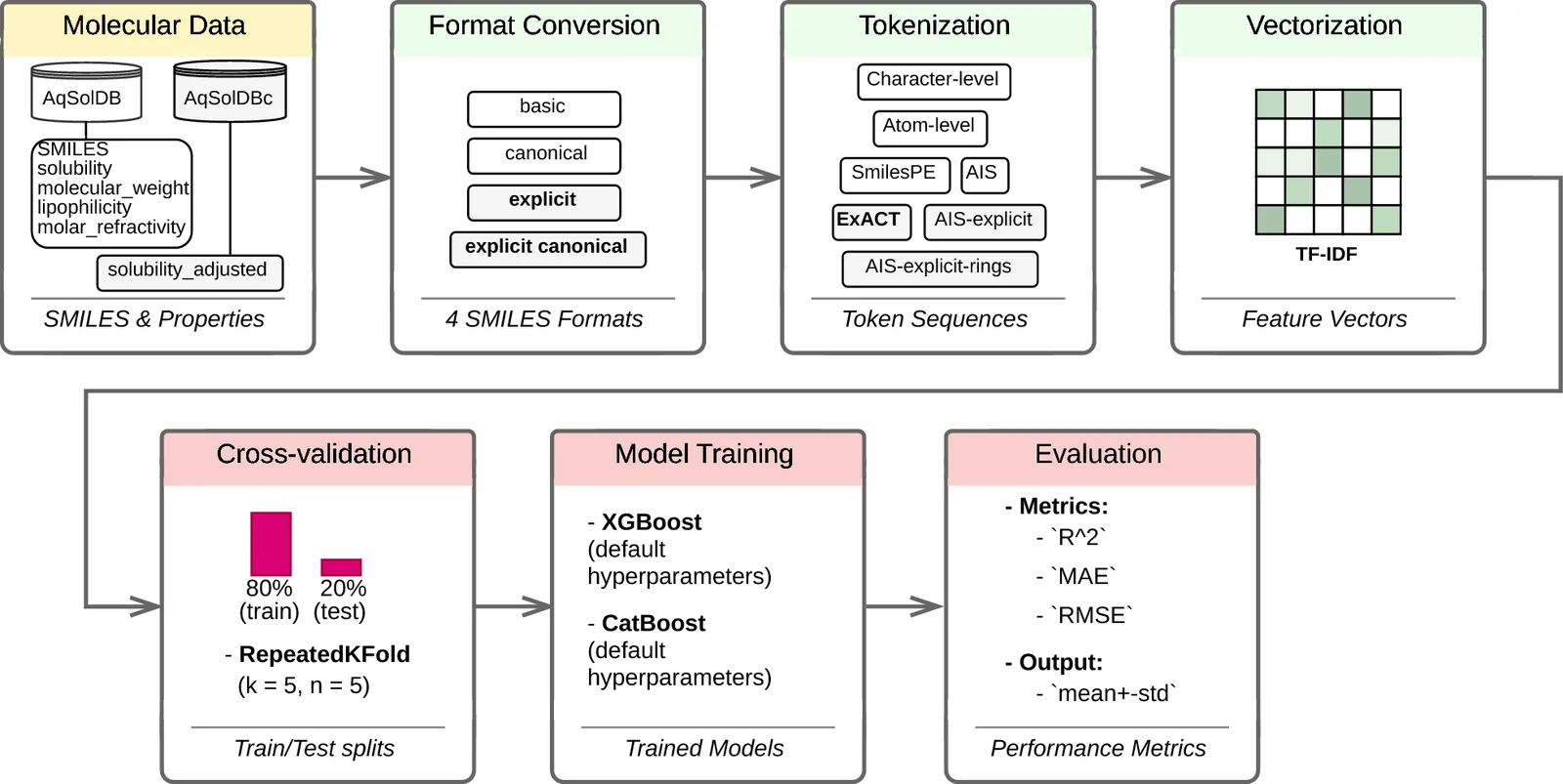
Process overview

The complete workflow is summarized in Fig. 1. Throughout all experiments, identical data partitions, preprocessing procedures, model configurations, and evaluation protocols were maintained to ensure reproducibility and enable a fair comparison among the investigated tokenization methods.

### Data

The predictive evaluation conducted in this study utilizes the widely referenced AqSolDB dataset [28], which comprises experimentally measured aqueous solubility values paired with corresponding SMILES representations for a diverse set of organic molecules. To further characterize the chemical diversity of the filtered AqSolDB dataset, distributions of selected structural and physicochemical descriptors are provided in Appendix D. In addition to the original dataset, we also employed a highly curated variant recently published to assess robustness of tokenization methods [33] which we used as a source for adjusted aqueous solubility measurements. Both aqueous solubility and adjusted aqueous solubility measurements are expressed in consistent *log*(*S*) units, enabling direct comparison across models. Aqueous solubility measurements encompasses a broad range of solubility values reflecting varying chemical classes, while the adjusted aqueous solubility measurements represent corrected and normalized records intended to mitigate measurement inconsistencies. Prior to model training, SMILES strings are validated for syntactic correctness, and molecules exhibiting invalid or ambiguous representations are excluded. During this validation step, only two SMILES samples in the original dataset were found to be syntactically invalid: *CC1=CC=C[NH++]([O-])[CH-]1* and *OC(=O)C1=C[NH++]([O-])[CH-]C=C1*. In both cases the nitrogen atom has an impossible valency of six, which is not allowed in SMILES grammar. As a result, no processing method that relies on correct SMILES grammar can work with these representations.

There is only one duplicate in AqSolDB: entries E-1289 and C-293 which both correspond to prostaglandin E2. While their stereochemical specifications differ, they represent the same molecular scaffold and have identical solubility values. To avoid duplicate sampling, we retained the stereochemically specified record (C-293) and removed E-1289.

**Fig. 2:**
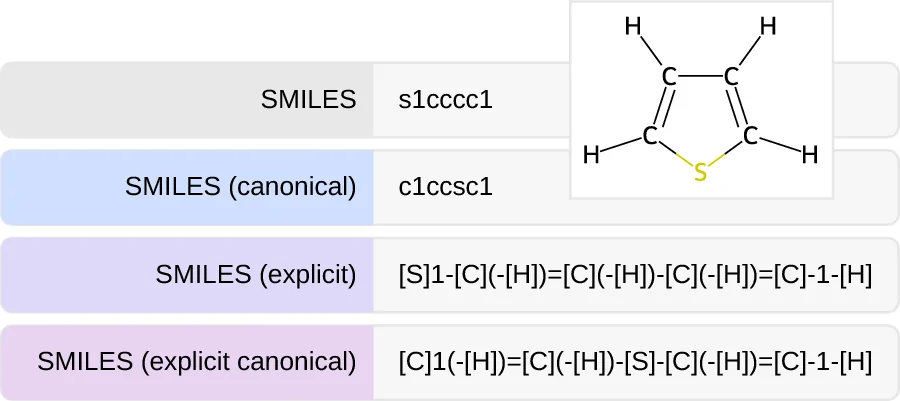
Used SMILES variations shown on an example from the AqSolDB dataset (thiophene)

**Fig. 3:**
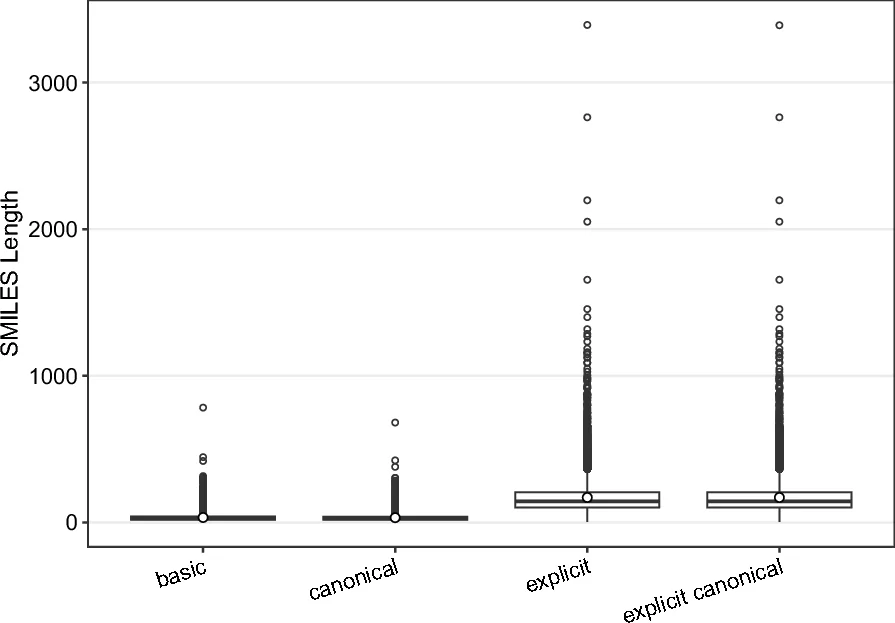
SMILES length boxplots across basic, canonical, explicit, and explicit canonical representations

For each retained molecule, four SMILES formats are generated: basic, canonical, explicit, and explicit canonical, to facilitate systematic comparison of tokenization methods across representational variants (Fig. 2). SMILES variants beyond the basic SMILES were generated using RDKit [34] and all generated variants conform to valid SMILES grammar and are syntactically correct. The distributions of sequence lengths for all four used SMILES variations for the AqSolDB dataset are illustrated in Fig. 3, providing a basic overview of representational complexity.

### Explicit SMILES tokenization: ExACT

**Fig. 4:**
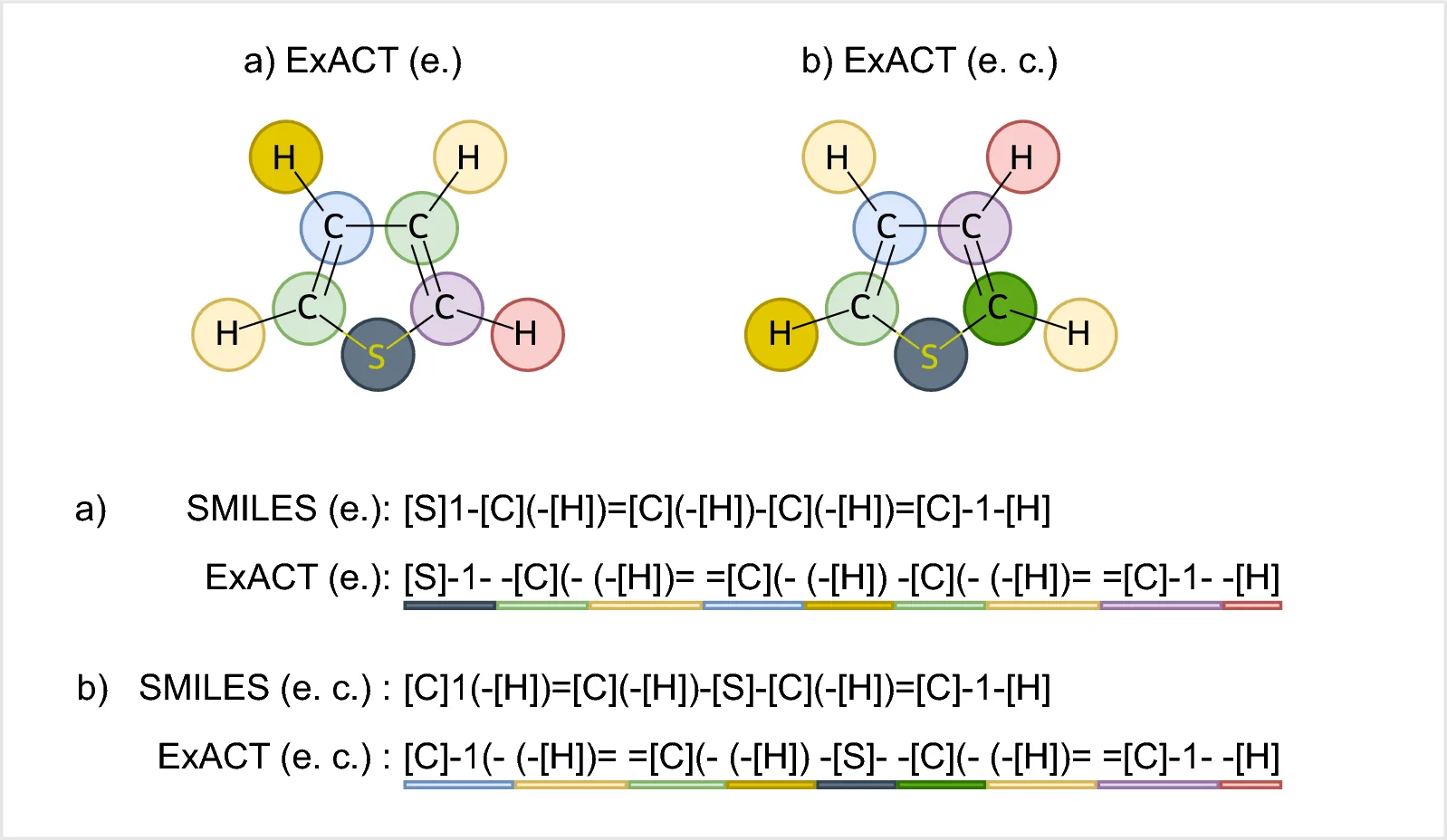
ExACT tokenization example for thiophene molecule. **a** Show ExACT applied to explicit SMILES, while** b** show ExACT applied to explicit canonical SMILES

The central methodological contribution of this work is a novel tokenization approach termed Explicit Atom level Context Tokenization (ExACT). In contrast to traditional tokenization methods that treat identical atom symbols as equivalent regardless of local environment, ExACT constructs tokens that encode both atom identity and local explicit chemical context derived from the explicit SMILES representation. This representation includes all hydrogen atoms and bond specifications, thereby supplying richer structural information for token creation. The ExACT algorithm systematically incorporates atom neighborhoods and bond connectivity into token definitions, yielding tokens that uniquely reflect atom position and environment within each molecule. An example of ExACT tokenization for a representative molecule is provided in Fig. 4, and the Algorithm 1 outlines the algorithmic steps for ExACT token generation. The proposed ExACT tokenization algorithm converts an explicit SMILES string *S* into a sequence of atom centered tokens while preserving structural information such as bonds, branching, and ring closures. The procedure first applies *SplitSMILESIntoParts(S)*, which segments the SMILES string into primitive components using a regular expression designed to identify atomic symbols, bracketed atoms, bond markers, digits representing ring indices, parentheses, and other SMILES control characters. The resulting sequence of parts is then processed sequentially to construct tokens. When a dot symbol is encountered, the current token is terminated and the dot is emitted as a separate token. When an atomic symbol or bracketed atom is encountered, a new token is created; if a previous token already exists, the operation MergeWithPreviousContext(current,p) is applied. This operation constructs the new token by combining the current atom symbol with the immediate structural context inherited from the preceding token, including bond symbols, branch delimiters, and ring-closure indicators that directly define the atom’s local connectivity. For example, a double bond (=), branch opening ((), or ring index (1) associated with the connection to the current atom is retained as part of the newly created token. In this way, ExACT preserves not only atom identity but also the explicit local bonding environment through which the atom participates in the molecular structure. After all parts have been processed, each token undergoes a normalization step using *NormalizeRingIndices(t)*, which adjusts the representation of ring closure indices by inserting explicit bond delimiters where required and removing redundant bond markers that may appear after closing parentheses. The final ordered list of tokens is then returned.

**Algorithm 1:**
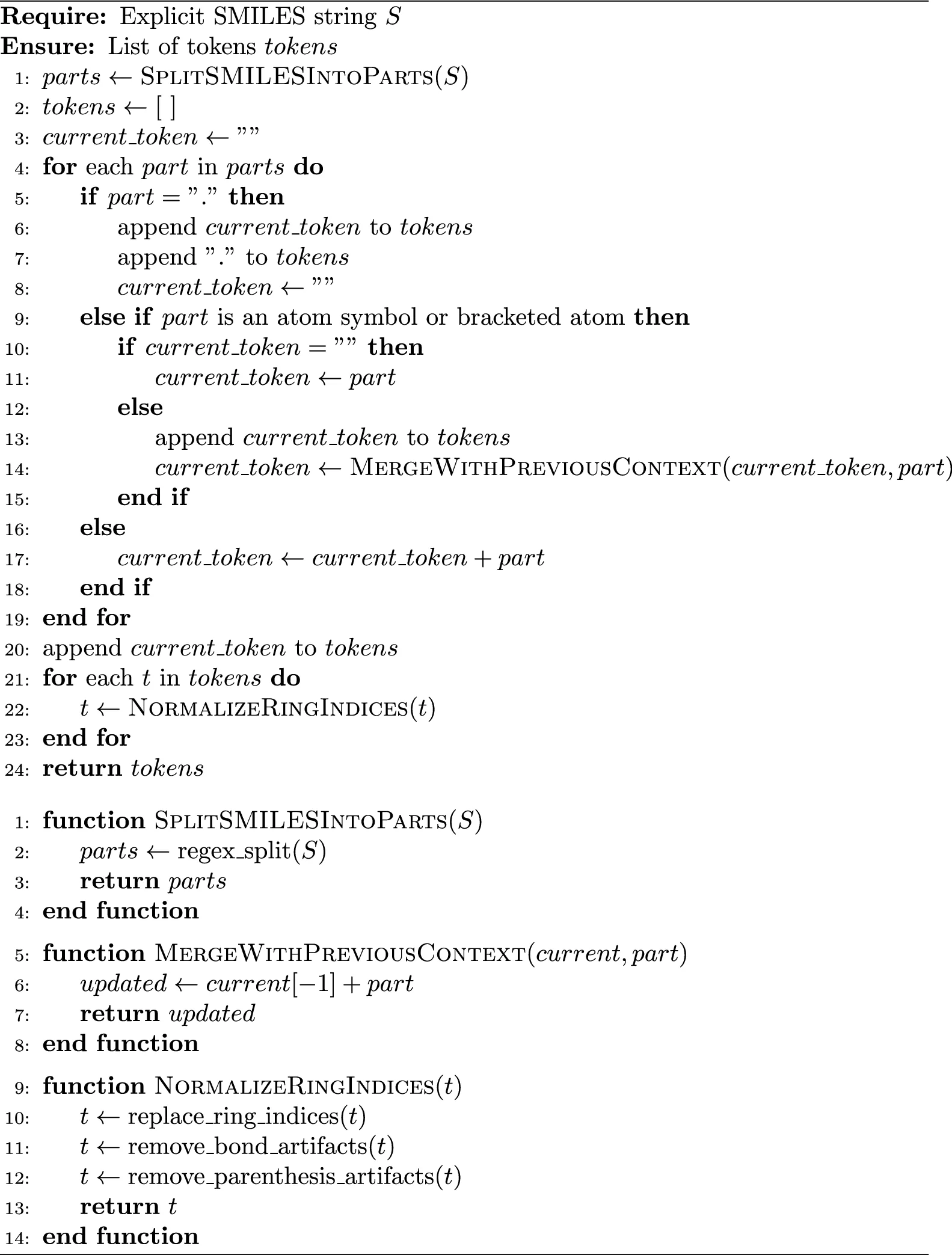
ExACT algorithm pseudocode

Figures 3 and 6 together show that canonicalization has little effect on the overall size of the molecular representation, but a noticeable impact on its token composition. As shown in Fig. 3, canonical and non-canonical SMILES exhibit very similar sequence-length distributions, indicating that canonicalization largely preserves the amount of structural information encoded for each molecule. Consistent with this observation, Fig. 6 demonstrates that ExACT token counts remain nearly unchanged between explicit and explicit canonical SMILES, confirming that canonicalization does not alter the overall representation granularity. Additionally, Appendix A demonstrates that token length distributions are largely preserved between the explicit and explicit canonical SMILES representations. However, the distribution of unique ExACT tokens shifts slightly after canonicalization, reflecting changes in token identity and frequency caused by the deterministic atom-ordering procedure. By enforcing a consistent traversal of molecular structures, canonicalization reduces representational variability while preserving molecular identity and information content. Consequently, canonicalized explicit SMILES provide a more statistically consistent basis for ExACT token generation, leading to a more uniform and structured token vocabulary across molecules.

**Fig. 5:**
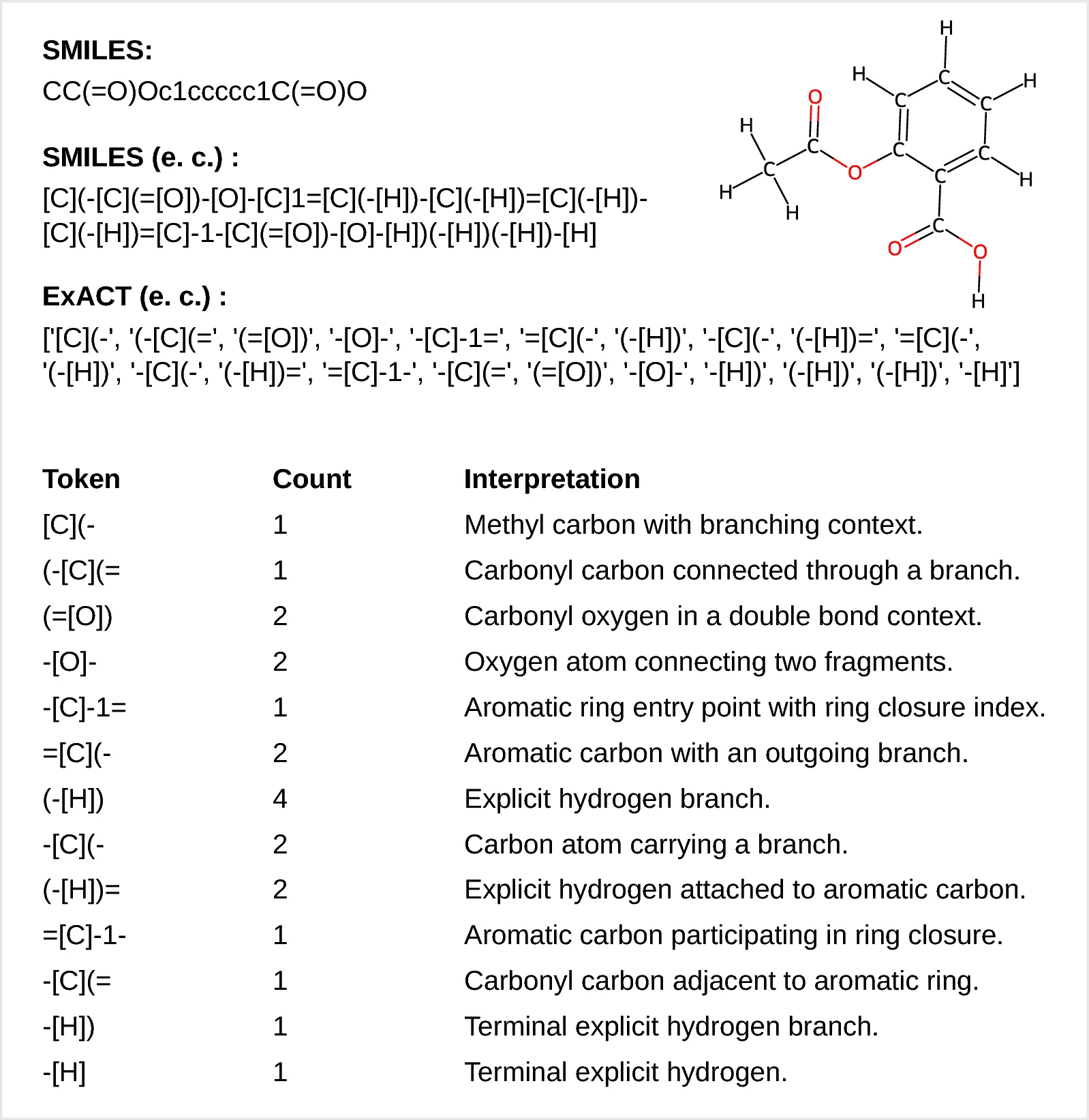
ExACT tokenization example for aspirin with interpreted tokens table

**Fig. 6:**
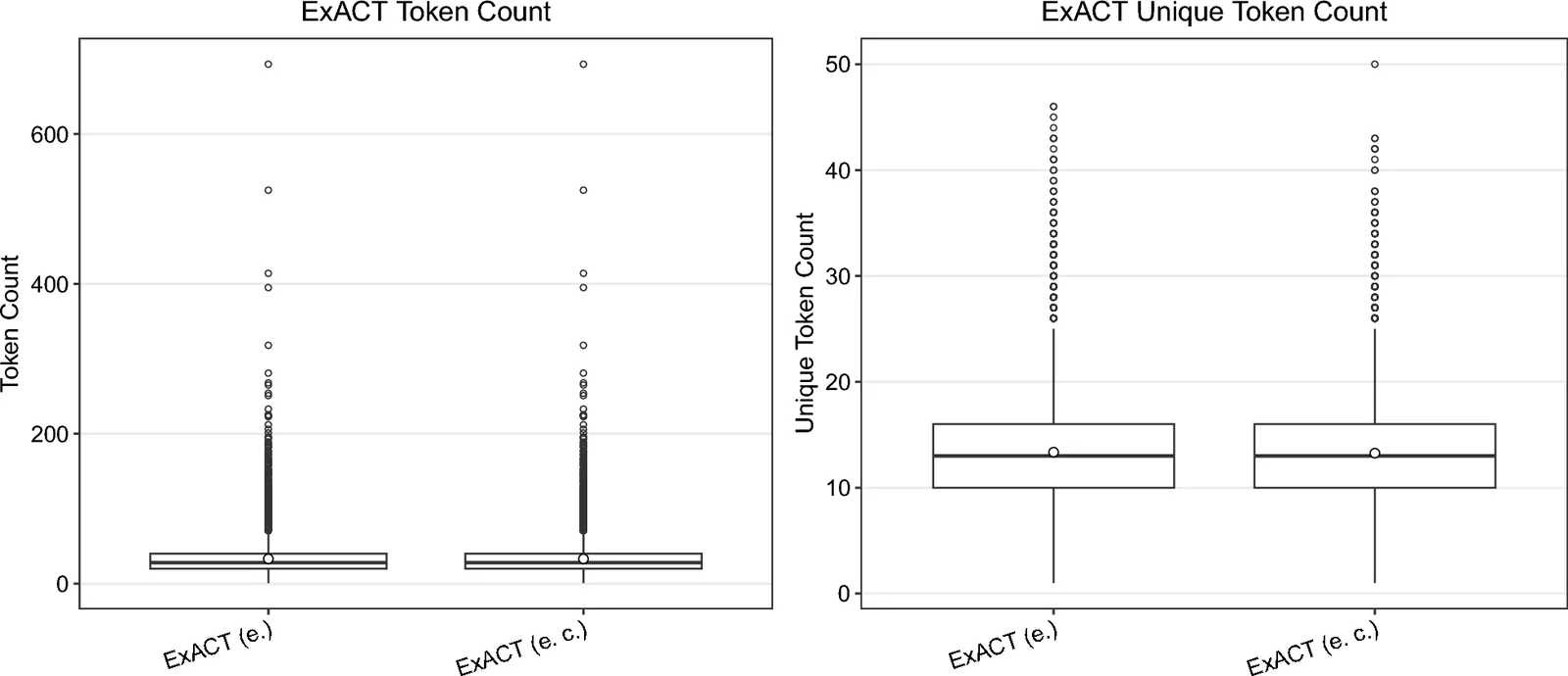
ExACT token count and unique token count boxplots using explicit and explicit canonical SMILES

Unlike data-driven subword tokenization methods that generate statistically optimized tokens with limited chemical meaning, ExACT tokens correspond to chemically interpretable local SMILES contexts. Each token preserves structural information such as atom identity, bond order, aromaticity, branching, ring closure, and explicit hydrogen environments. Consequently, the generated tokens remains human-interpretable and can be directly mapped to recognizable chemical motifs. In Fig. 5 we demonstrate ExACT tokenization method on aspirin molecule and provide token interpretations. For example, the token (=[O]) represents a carbonyl oxygen environment with branching context, -[O]- denotes an oxygen atom connecting two molecular tokens, and -[C]-1= identifies an aromatic ring entry point associated with a ring-closure index. This context-preserving design facilitates mechanistic interpretation of model features while maintaining robustness across diverse molecular structures.

Finally, a note about chosen terminology. While the resulting tokens are utilized as fragments for descriptor construction in the current study, the method was specifically designed to produce context-aware token sequences that are directly compatible with language model based molecular representation learning. Retaining the term tokenization therefore reflects both the nature of the transformation itself and the broader applicability of ExACT within molecular language processing frameworks. In the present study these tokens are subsequently transformed into fragment-based descriptors via TF-IDF encoding (Fig. 6).

### Models and rationale

In this study, two established machine learning regression models were selected for evaluation: Extreme Gradient Boosting (XGB) and CatBoost regression. Both methods are ensemble learning algorithms based on gradient boosted decision trees and have demonstrated strong predictive performance across a wide range of structured data applications, including molecular property prediction tasks. Their use enables systematic assessment of how explicit SMILES tokenization influences predictive performance across distinct molecular property prediction tasks while minimizing confounding effects arising from substantially different model architectures.

The XGB model was selected due to its strong empirical performance, computational efficiency, and ability to model complex nonlinear relationships between molecular representations and target properties. XGBoost incorporates regularization mechanisms, efficient tree construction strategies, and optimized learning procedures that help reduce overfitting while maintaining high predictive accuracy. Furthermore, its widespread adoption in cheminformatics and molecular property prediction studies facilitates direct comparison with existing literature and established benchmarking practices. The model is particularly well suited for handling heterogeneous feature spaces derived from different molecular tokenization strategies and molecular descriptors.

CatBoost regression was included as a complementary gradient boosting approach due to its robustness, strong generalization capability, and native support for categorical feature representations. Unlike XGBoost, which generally requires categorical variables to be transformed through external encoding procedures before training, CatBoost was specifically designed to process categorical information through internal encoding mechanisms and ordered boosting strategies. Although the present study employed TF-IDF vectorization for all tokenized representations and therefore did not utilize CatBoost’s native categorical feature processing pipeline, the model remains particularly relevant for token based molecular representations because SMILES tokens and molecular fragments originate as categorical entities. Consequently, CatBoost provides a useful point of comparison to XGBoost, as its underlying design is inherently suited to learning from categorical feature structures. In addition, CatBoost employs ordered boosting and advanced regularization techniques that mitigate prediction shift and reduce overfitting during training, resulting in stable learning behavior and competitive predictive performance across diverse datasets. These characteristics make CatBoost well suited for evaluating the influence of alternative tokenization strategies and fragment-based molecular representations on molecular property prediction tasks.

A deliberate methodological decision was made not to pursue transformer based deep learning models in the present work. The primary objective of this study is to compare tokenization methods under controlled experimental conditions, isolating the effect of representation and tokenization on model performance. Tree based machine learning regressors provide a stable and well established framework that permits clearer attribution of performance differences to the tokenization methods themselves rather than to model architecture complexity, representation learning mechanisms, or optimization dynamics. Furthermore, transformer models typically require substantially larger datasets, extensive computational resources, and careful hyperparameter optimization to fully realize their predictive potential. Such requirements can complicate comparative analyses when the primary research objective is methodological evaluation of tokenization approaches rather than maximizing predictive performance.

The selected modeling framework was evaluated on two molecular property prediction tasks: aqueous solubility and molecular weight prediction. Aqueous solubility, in contrast, represents a substantially more challenging physicochemical property influenced by a combination of structural characteristics and intermolecular interactions. Experimental solubility measurements are inherently associated with greater variability and uncertainty than calculated molecular descriptors, making prediction considerably more difficult. Molecular weight was intentionally included as a baseline target because it is a deterministic property that can be calculated directly from molecular structure without experimental uncertainty.

By extending the evaluation from molecular weight to aqueous solubility, it becomes possible to determine whether tokenization strategies that effectively capture basic structural information also retain their utility when applied to a more complex and practically relevant property prediction task. Evaluating tokenization methods across these two properties enables assessment of their effectiveness in capturing both complex physicochemical relationships and more directly encoded structural information. This dual task evaluation provides a broader perspective on the suitability and generalizability of different tokenization strategies for molecular machine learning applications.

## Results

The results are presented through three analyses. First, predictive performance analysis evaluates the influence of SMILES representation and tokenization strategy on aqueous solubility and molecular weight prediction using XGBoost and CatBoost regressors. Second, statistical significance analysis employs Tukey’s HSD test to determine whether the performance differences between the best-performed ExACT approach and other approaches are statistically meaningful. Third, efficiency analysis examines the computational cost and feature space dimensionality associated with each preprocessing approach, providing insight into the trade-off between predictive accuracy and computational complexity. Finally, best-performed ExACT approach is compared with results reported in contemporary studies to position its predictive performance within the current state-of-the-art.

### Predictive performance analysis

The predictive performance of the evaluated tokenization methods across multiple SMILES representation variants and machine learning models is summarized in Tables 1 and 2. Table 1 reports results obtained for aqueous solubility property, whereas Table 2 reports results on the molecular weight property. For each appropriate combination of SMILES input format (basic, canonical, explicit, explicit canonical) and tokenization method (character-level, atom-level, SmilesPE, atom-in-SMILES, ExACT and both atom-in-SMILES-explicit variations), performance metrics including coefficient of determination ($R^2$), mean absolute error (*MAE*), and root mean squared error (*RMSE*) are presented for each regression model. These metrics provide comprehensive assessment of model fit, average error magnitude, and sensitivity to large errors respectively.

When it comes to aqueous solubility, across all evaluated models a consistent positively correlated relationship is observed between representational explicitness and predictive performance. Transitioning from basic to canonical SMILES yields marginal improvements, while the shift from implicit to explicit representations produces substantial and systematic gains across all tokenization strategies. This pattern is reflected in increased coefficients of determination and corresponding reductions in *MAE* and *RMSE*, indicating that explicit representations encode chemically relevant information that is directly exploitable by sequence based learning pipelines. This effect is not restricted to a single model implementation. Although both CatBoost and XGB belong to the gradient boosting family, they employ different strategies for tree construction, feature processing, and model optimization. The observation of similar performance trends across both algorithms indicates that the improvements are primarily attributable to the molecular representation itself rather than to model specific characteristics. This consistency suggests that increasing representational explicitness enhances the information content of the input features in a manner that is robust across different boosting based learning frameworks.

For molecular weight property, atom-level and character-level on basic SMILES format produce the best results for XGB model and very competitive results for CatBoost. This was somewhat expected since molecular weight is an additive property that depends almost entirely on the presence and counts of atoms in a molecule. In this case, a simpler representation offers a straightforward way of calculating the target variable using a weighted sum, while adding more context does not necessarily improve the predictive power of the model. Despite this fact, ExACT tokenization method does produce the best overall results when using the CatBoost model. This proves that the ExACT tokenization method can still be effectively applied even on simple target variables that can be calculated in a straightforward manner, as well as on more complex properties such as aqueous solubility, whose accurate determination relies on carefully conducted experimental measurements. ExACT’s architecture, which constructs atom centered tokens from explicit local neighborhoods, appears particularly robust to noise and heterogeneity in experimental data. This suggests that explicit atom level contextualization functions as a form of structural regularization, stabilizing learning in the presence of label noise and measurement variability.

SmilesPE exhibits weak performance, despite its substantially greater algorithmic sophistication. Although SmilesPE leverages a data-driven subword vocabulary pretrained on a large-scale corpus of approximately 1.7 million SMILES strings from the ChEMBL database, its tokens remain determined by frequency of recurrent substrings, which is not a chemically grounded approach. This limitation constrains its predictive capacity relative to chemically contextualized tokenization methods. Context-enriched tokenization methods form a distinct upper performance tier. AIS outperforms traditional tokenization methods and fingerprint methods in aqueous solubility prediction task, confirming prior findings that embedding local chemical environment information into tokens enhances predictive modeling. For molecular weight prediction, AIS falls short and cannot produce competitive results. The introduction of explicitness further amplifies the effect of embedding local chemical environment. Both ExACT and two AIS-explicit variations surpass all non-explicit tokenization strategies across most experimental configurations, demonstrating that explicit hydrogen specification and bond detail materially improve the discriminative power of token sequences. These results indicate that tokenization quality is not solely a function of algorithmic sophistication but is fundamentally constrained by the information density of the underlying representation. Explicit SMILES increase the available chemical signal at the representation level, enabling tokenization methods to construct more informative, less degenerate, and more chemically interpretable token vocabularies.

Performance gains are observed not only in novel methods such as ExACT but also when applied as a systematic upgrade to existing approaches, as demonstrated by two AIS-explicit variations. Explicit SMILES representations expose chemically meaningful primitives, including hydrogen bonding capacity, saturation states, and precise bond order relationships, which are otherwise implicit or abstracted. When this information is preserved at the representation level, tokenization methods can encode it directly into discrete tokens, enabling conventional machine learning models to exploit chemical semantics without reliance on graph based representations or neural message passing architectures.

Additionally, explicit tokenization based approaches outperform traditional molecular fingerprint representations. This is a critical structural result, as it demonstrates that sequence based representations, when sufficiently explicit and context-enriched, can match the predictive capacity of chemically engineered feature spaces while retaining interpretability, modularity, and compatibility with standard machine learning pipelines. This finding challenges the prevailing assumption that high fidelity molecular property prediction requires either handcrafted descriptors or graph based neural architectures. Instead, the results establish explicit textual representations combined with chemically informed tokenization as a viable and competitive paradigm for molecular property modeling.

**Table 1:** Results for aqueous solubility property

Method	XGB			CatBoost		
	$R^2$	*MAE*	*RMSE*	$R^2$	*MAE*	*RMSE*
Character	.710±.016	.922±.018	1.273±.033	.725±.016	.902±.021	1.240±.035
Character (c.)	.695±.017	.951±.020	1.308±.034	.710±.017	.935±.021	1.273±.036
Character (e.)	.723±.015	.889±.018	1.244±.032	.740±.012	.874±.015	1.208±.029
Character (e. c.)	.724±.014	.889±.018	1.244±.032	.741±.013	.871±.016	1.205±.030
Atom	.711±.017	.930±.020	1.271±.036	.723±.014	.913±.017	1.245±.031
Atom (c.)	.689±.017	.967±.022	1.321±.034	.704±.015	.950±.018	1.287±.029
Atom (e.)	.720±.015	.900±.016	1.251±.030	.730±.014	.894±.014	1.230±.030
Atom (e. c.)	.723±.015	.896±.018	1.245±.033	.733±.013	.891±.014	1.223±.029
SmilesPE	.586±.016	1.158±.021	1.522±.031	.611±.016	1.129±.023	1.476±.031
SmilesPE (c.)	.567±.014	1.193±.022	1.557±.029	.588±.013	1.172±.021	1.520±.027
SmilesPE (e.)	.718±.015	.910±.017	1.257±.033	.728±.013	.901±.015	1.233±.029
SmilesPE (e. c.)	.719±.018	.905±.020	1.255±.039	.732±.014	.892±.014	1.225±.029
AIS (c.)	$\mathbf {.737\pm .015}$	$\mathbf {.870\pm .018}$	$\mathbf {1.213\pm .035}$	.754±.012	$\mathbf {.848\pm .016}$	1.175±.028
AIS-e (e. c.)	.726±.014	.892±.016	1.239±.033	.744±.012	.868±.014	1.197±.029
AIS-er (e. c.)	.719±.014	.906±.018	1.254±.033	.738±.011	.879±.014	1.212±.028
ExACT (e.)	.725±.018	.896±.022	1.240±.038	.749±.015	.860±.018	1.186±.033
ExACT (e. c.)	.732±.015	.887±.020	1.225±.035	$\mathbf {.756\pm .013}$	.850±.017	$\mathbf {1.170\pm .029}$
MACCs	.724±.012	.891±.020	1.244±.029	.737±.012	.876±.018	1.213±.029
Morgan (r=2)	.660±.013	1.012±.017	1.381±.029	.672±.011	.999±.013	1.355±.025

*e.* denotes explicit SMILES, *c.* canonical SMILES and *e. c.* explicit canonical SMILES. AIS-e corresponds to AIS-explicit, and AIS-er to AIS-explicit-rings methods defined in Appendix B

**Table 2:** Results for molecular weight property

Method	XGB			CatBoost		
	$R^2$	*MAE*	*RMSE*	$R^2$	*MAE*	*RMSE*
Character	.780±.064	33.1±1.5	85.7±18.7	.800±.072	32.1±1.5	81.6±21.8
Character (c.)	.656±.081	46.1±1.9	107.7±22.3	.713±.079	45.2±1.6	98.4±22.5
Character (e.)	.707±.081	38.5±2.1	99.3±22.6	.736±.086	38.3±1.7	94.1±24.0
Character (e. c.)	.698±.072	39.3±1.8	100.9±21.1	.732±.082	39.0±1.3	94.9±23.2
Atom	$\mathbf {.786\pm .069}$	33.5±1.6	$\mathbf {84.6\pm 20.5}$	.792±.079	33.2±1.7	83.3±23.4
Atom (c.)	.643±.074	46.9±1.7	109.8±21.5	.689±.075	47.5±1.6	102.3±21.9
Atom (e.)	.678±.086	41.0±2.2	104.1±23.2	.710±.086	41.9±1.9	98.8±23.6
Atom (e. c.)	.678±.089	41.8±2.2	104.0±23.7	.710±.085	42.7±1.8	98.8±23.4
SmilesPE	.705±.102	49.1±2.1	99.4±26.0	.701±.099	49.8±2.0	100.0±25.4
SmilesPE (c.)	.573±.086	57.9±2.0	120.0±22.7	.606±.081	58.0±1.9	115.1±21.5
SmilesPE (e.)	.679±.078	41.8±1.9	104.0±22.1	.719±.085	42.0±1.9	97.2±23.7
SmilesPE (e. c.)	.687±.088	41.9±1.9	102.5±23.5	.716±.086	42.8±1.8	97.7±23.8
AIS (c.)	.665±.074	42.4±1.7	106.3±21.2	.731±.084	41.4±1.5	95.2±23.8
AIS-e (e. c.)	.740±.094	33.5±2.2	93.3±25.5	.781±.085	32.2±1.7	85.7±24.7
AIS-er (e. c.)	.734±.094	34.1±2.0	94.5±25.5	.778±.087	32.7±1.5	86.2±25.0
ExACT (e.)	.722±.085	30.6±1.8	96.7±23.9	.801±.091	25.2±1.4	81.4±26.3
ExACT (e. c.)	.738±.092	$\mathbf {29.5\pm 1.8}$	93.6±25.1	$\mathbf {.803\pm .089}$	$\mathbf {25.1\pm 1.4}$	$\mathbf {81.0\pm 25.8}$
MACCs	.629±.106	46.9±1.9	111.0±19.7	.665±.081	45.5±1.7	105.9±20.2
Morgan (r=2)	.583±.080	54.6±2.0	118.4±21.3	.613±.074	55.2±1.7	114.3±21.5

*e.* denotes explicit SMILES, *c.* canonical SMILES and *e. c.* explicit canonical SMILES. AIS-e corresponds to AIS-explicit, and AIS-er to AIS-explicit-rings methods defined in Appendix B

### Statistical significance analysis

**Fig. 7:**
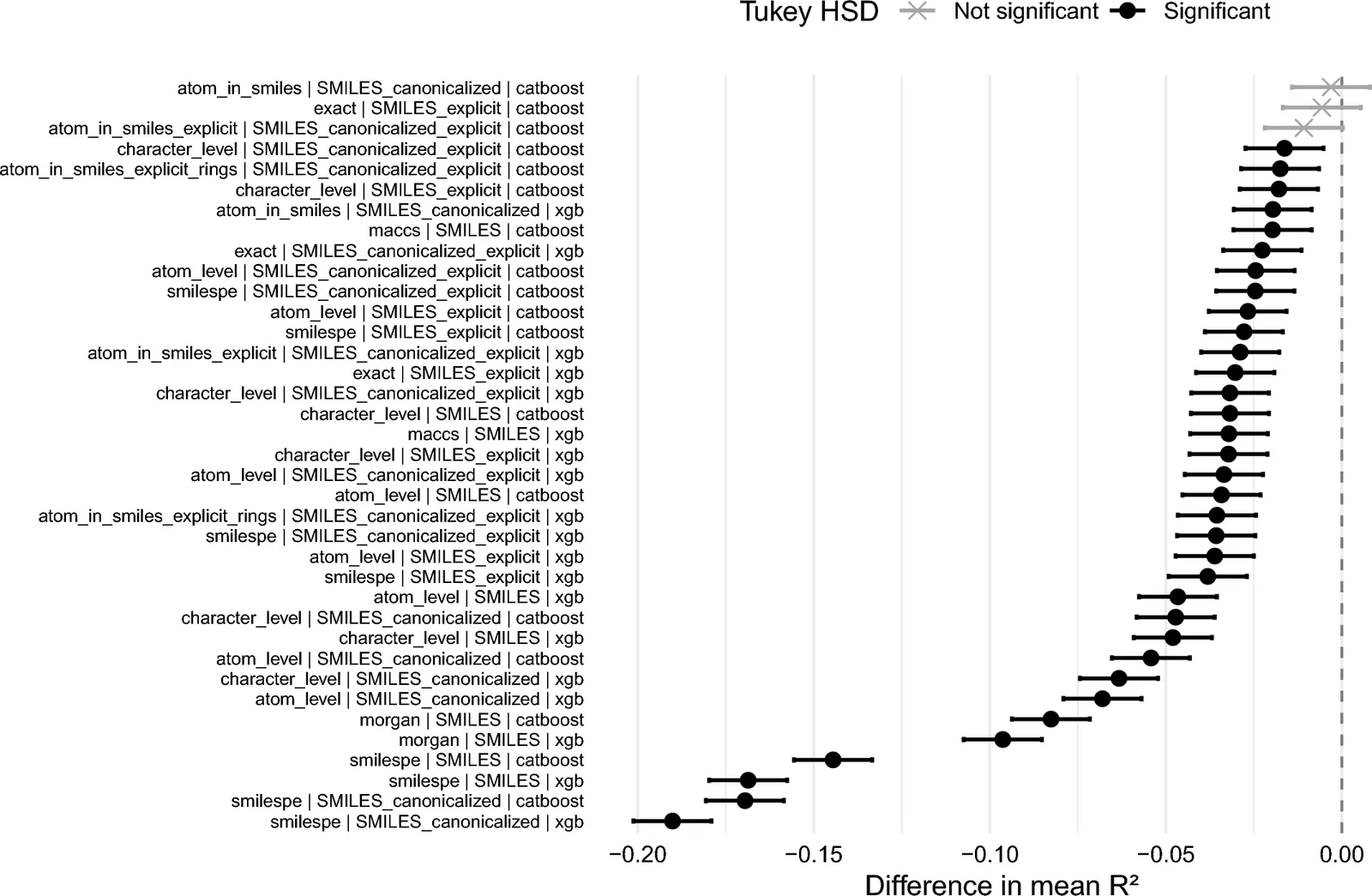
Tukey’s Honest Significant Difference (HSD) post-hoc comparison of the ExACT best-performing pipeline against all other evaluated pipelines

Recent studies have emphasized that small differences in predictive metrics should be interpreted cautiously and supported by appropriate statistical testing, particularly when comparing multiple machine learning models evaluated using repeated validation procedures [35]. To assess whether the observed performance differences among the evaluated pipelines were statistically meaningful, a post hoc Tukey’s Honestly Significant Difference (HSD) analysis was performed on the repeated cross validation results. Although Tukey’s HSD is commonly used for all pairwise comparisons, the primary objective of this study was not to establish a complete ranking of all evaluated pipelines, but rather to determine whether the proposed ExACT based approach significantly outperformed alternative representations. Consequently, the analysis was centered on a single reference pipeline consisting of the CatBoost regressor trained on ExACT features derived from canonicalized explicit SMILES representations. This approach was selected as the reference because it achieved the highest overall predictive performance for aqueous solubility prediction. The Tukey analysis compared the reference pipeline against all remaining approaches while controlling the family-wise error rate associated with multiple comparisons. Such a targeted comparison provides a more direct assessment of the practical research question, namely whether the proposed methodology offers a statistically significant advantage over existing alternatives, rather than merely identifying differences among all possible pairs of methods. The results indicate that only three alternative approaches were statistically indistinguishable from the reference model. The first was the original Atom-in-SMILES (AIS) representation, which has previously demonstrated strong predictive performance. However, ExACT achieved comparable predictive accuracy while requiring substantially lower computational time and a lower dimensional feature space. The second approach was ExACT applied to noncanonicalized explicit SMILES strings. The similarity in performance between these variants suggests that molecular canonicalization exerts only a minor influence on the ExACT representation in this prediction task, indicating robustness of the proposed tokenization strategy to atom ordering. The third statistically equivalent approach was the explicit AIS variant. Although competitive in predictive performance, this representation produced substantially higher dimensional feature vectors and required the longest computational time among the evaluated methods. Collectively, these findings indicate that ExACT achieves a favorable balance between predictive accuracy, computational efficiency, and feature space complexity while remaining among the statistically best performing approaches for aqueous solubility prediction (Fig. 7).

### Efficiency analysis

Table 3 reports number of features (*n*), time to compute XGB model (*t* (XGB)) and time to compute CatBoost model (*t* (CatBoost)) for each preprocessing method and property. SmilesPE shows a drastic reduction in vocabulary size when explicitness is applied, which also reflects on time to compute regardless of used model. ExACT produces smaller feature space than standard AIS, offering a compact and more efficient representation. This also reflects on time to compute. In contrast, AIS-explicit and AIS-explicit-rings substantially increase feature space compared to AIS, improving context encoding but introducing much higher dimensionality. Since TF-IDF encoding maps each token to an individual feature dimension, larger number of features result in higher-dimensional feature spaces. This may increase sparsity and computational cost, potentially affecting model efficiency and generalization. MACCs and Morgan fingerprints both have fixed number of features and take substantial amount of time to compute in comparison to other preprocessing methods.

**Table 3:** Number of features (*n*) and time to compute (*t*) per used preprocessing method

Method	*n*	Solubility		Molecular weight	
		*t* (XGB)	*t* (CatBoost)	*t* (XGB)	*t* (CatBoost)
Character	62±1	0.26±0.01	1.21±0.02	0.26±0.01	1.23±0.02
Character (c.)	61±1	0.26±0.01	1.19±0.03	0.26±0.01	1.21±0.02
Character (e.)	58±1	0.40±0.01	1.30±0.02	0.41±0.01	1.31±0.01
Character (e. c.)	59±1	0.40±0.01	1.31±0.01	0.40±0.01	1.31±0.02
Atom	187±2	0.29±0.00	1.31±0.03	0.30±0.00	1.30±0.02
Atom (c.)	188±3	0.29±0.01	1.27±0.02	0.29±0.01	1.29±0.02
Atom (e.)	148±2	0.42±0.01	1.34±0.01	0.43±0.01	1.36±0.02
Atom (e. c.)	149±2	0.42±0.01	1.36±0.02	0.43±0.01	1.36±0.02
SmilesPE	1991±11	0.80±0.09	3.87±0.07	0.81±0.02	4.03±0.04
SmilesPE (c.)	2319±12	0.91±0.10	3.66±0.02	0.95±0.02	3.85±0.02
SmilesPE (e.)	180±3	0.40±0.05	1.41±0.02	0.40±0.01	1.44±0.02
SmilesPE (e. c.)	180±3	0.40±0.03	1.43±0.01	0.40±0.01	1.44±0.02
AIS (c.)	875±8	3.15±0.04	5.29±0.05	3.21±0.02	5.45±0.04
AIS-e (e. c.)	2093±16	3.81±0.03	6.98±0.07	3.87±0.06	7.09±0.04
AIS-er (e. c.)	3369±24	5.84±0.04	7.95±0.04	6.08±0.06	8.03±0.05
ExACT (e.)	671±7	0.76±0.01	2.74±0.02	0.76±0.01	2.76±0.03
ExACT (e. c.)	654±8	0.75±0.01	2.61±0.01	0.76±0.01	2.70±0.03
MACCs	167±0	3.57±0.04	5.14±0.02	3.49±0.02	5.19±0.03
Morgan (r=2)	2048±0	3.57±0.03	5.94±0.13	3.59±0.03	5.85±0.14

*e.* denotes explicit SMILES, *c.* canonical SMILES and *e. c.* explicit canonical SMILES. AIS-e corresponds to AIS-explicit, and AIS-er to AIS-explicit-rings

### Comparison with contemporary studies

Recent aqueous solubility prediction studies have increasingly shifted toward deep learning architectures, particularly transformer based models, graph neural networks, and hybrid approaches that combine multiple molecular representations. The Transformer-CNN framework introduced by [36] employs a transformer encoder trained on SMILES strings followed by convolutional neural network layers for property prediction. Using AqSolDB dataset, the authors reported a *RMSE* of 0.79±0.01. The approach relies on learned molecular embeddings obtained from transformer based sequence modeling and represents one of the earliest successful applications of transformer architectures in QSAR modeling. More recently, [37] proposed a physics informed neural network (PINN) for AqSolDB that integrates thermodynamic constraints directly into the loss function. The model combines neural network learning with physically interpretable solvation energy components and was evaluated using a random train test split on the complete AqSolDB dataset. The reported performance reached an *RMSE* of 0.36 and an $R^2$ of 0.751, substantially exceeding the performance of conventional machine learning baselines reported in the same study. In comparison, the best performing configuration identified in the present work, ExACT tokenization applied to canonicalized explicit SMILES and coupled with CatBoost regression, achieved an $R^2$ of 0.756±0.013 and an RMSE of 1.170±0.029 using a repeated 5 × 5 cross validation protocol. Direct numerical comparison should be interpreted cautiously because the cited studies employed different datasets, data partitions, and evaluation methodologies. Nevertheless, the comparison highlights an important observation. While current state of the art research increasingly relies on sophisticated transformer, graph neural network, and physics informed architectures, competitive predictive performance can still be achieved through advances in molecular representation and tokenization. The results suggest that chemically informed preprocessing remains a valuable and complementary direction to the ongoing development of increasingly complex deep learning models.

## Conclusions and future work

This study investigated the influence of explicitness in multiple forms of SMILES representation on tokenization quality and predictive performance for aqueous solubility. The results demonstrate that incorporating explicit chemical detail into tokenization systematically improves model performance across established evaluation metrics for a complex problem such as aqueous solubility.

Tokenization approaches that encode richer chemical context consistently outperformed traditional representations. The proposed ExACT (Explicit Atom-level Context Tokenization) method achieved the highest $R^2$ and *RMSE* on the aqueous solubility property prediction task, and highest $R^2$, *MAE* and *RMSE* on molecular weight property prediction task. These findings support the central premise that explicitness introduces meaningful chemical context into sequence representations and that this additional information can be effectively exploited by machine learning models without relying on more complex deep learning architectures such as graph neural networks.

More broadly, the results indicate that making chemical information explicit at the representation level rather than relying on increasingly complex models to infer it implicitly, offers a practical way to improve predictive performance. This is further supported by the observation that explicit SMILES representations combined with context-aware tokenization can outperform traditional molecular fingerprinting approaches.

Chemically explicit tokenization decomposes molecules into well defined context-aware atomic and fragment-level units, enabling models to learn from localized chemical environments rather than relying solely on molecular structure representations. Such representations may be particularly valuable in drug discovery and ADMET prediction, where subtle differences in local chemical context can strongly influence molecular properties and biological activity. Because the resulting tokens correspond to chemically meaningful environments, their learned importance can be interpreted and compared across molecules and biological targets, helping to explain how local structural context influences molecular properties and drug–target interactions. This is consistent with recent efforts emphasizing interpretable machine learning approaches that identify chemically meaningful features responsible for model predictions rather than relying solely on predictive performance metrics [38].

Several directions for future research emerge from this work. The present evaluation focused exclusively on aqueous solubility prediction using the AqSolDB dataset and its curated variant; broader validation across additional physicochemical and biological endpoints is therefore needed. Extending the analysis to tasks such as bioactivity prediction, ligand-based drug discovery, toxicity prediction, pharmacokinetic property prediction, and related molecular modeling applications would help determine the general applicability of explicit tokenization. Particular emphasis may be placed on large-scale experimental bioactivity datasets such as ChEMBL and related repositories, where the ability of explicit context-based tokenization to support prediction across diverse biological targets and assay types can be systematically evaluated.

Different prediction tasks often depend on different types of information encoded within molecular structures, a challenge that is well recognized in computational biology and cheminformatics. As a result, a representation that performs well for one problem may not be optimal for another, highlighting the importance of including methods such as ExACT when benchmarking molecular machine learning approaches across diverse real-world applications.

Another promising direction involves integrating explicit tokenization into pre-training and transfer learning frameworks, which may further improve model robustness and facilitate cross-dataset knowledge transfer. Hybrid approaches that combine explicit sequence representations with graph-based or three-dimensional structural descriptors also merit investigation, as such combinations may capture complementary aspects of molecular structure [39]. Moreover, analytical approaches that relate token contributions to specific chemical substructures could provide mechanistic insight and enhance interpretability. Finally, future work will investigate ExACT within a transformer-based molecular language model, allowing the proposed context-aware tokens to be evaluated directly in architectures where tokenization is a fundamental component of molecular representation learning.

In summary, explicit SMILES representations coupled with appropriate tokenization strategies provide clear advantages for sequence-based molecular property prediction. By producing representations with chemically meaningful local environments, explicit tokenization may improve the modeling of structure–property relationships and therefore supports a wide range of applications in molecular design and drug discovery. The present findings therefore suggest that chemically explicit tokenization represents not only as a methodological advancement in cheminformatics, but as a practical approach for prioritizing compounds and improving the overall efficiency of drug discovery workflows.

## Supplementary Information

Supplementary material 1.

## Data Availability

All code used to implement the ExACT and AIS-explicit methods can be found in the designated code repository: https://gitlab.com/d-begusic/exact. This also includes the script for generating comparison results and visualizations of tokenization methods on the used AqSolDB and AqSolDBc datasets as well as datasets themselves.
