# Supplementary material for: Explicitness in SMILES representation via ExACT: improved tokenization for aqueous solubility prediction
Source: J Cheminform. 2026 Aug 4;18:151. doi: 10.1186/s13321-026-01263-w (PMC13721668; doi:10.1186/s13321-026-01263-w)
Supplement: Supplementary file 1 [file 13321_2026_1263_MOESM1_ESM.pdf]

Appendix A ExACT token length distributions

As shown in Figure A1, token length distributions remain largely consistent between AqSolDB and AqSolDBc, indicating that canonicalization does not substantially alter average token length, but produces a slightly flatter distribution due to deterministic atom ordering.

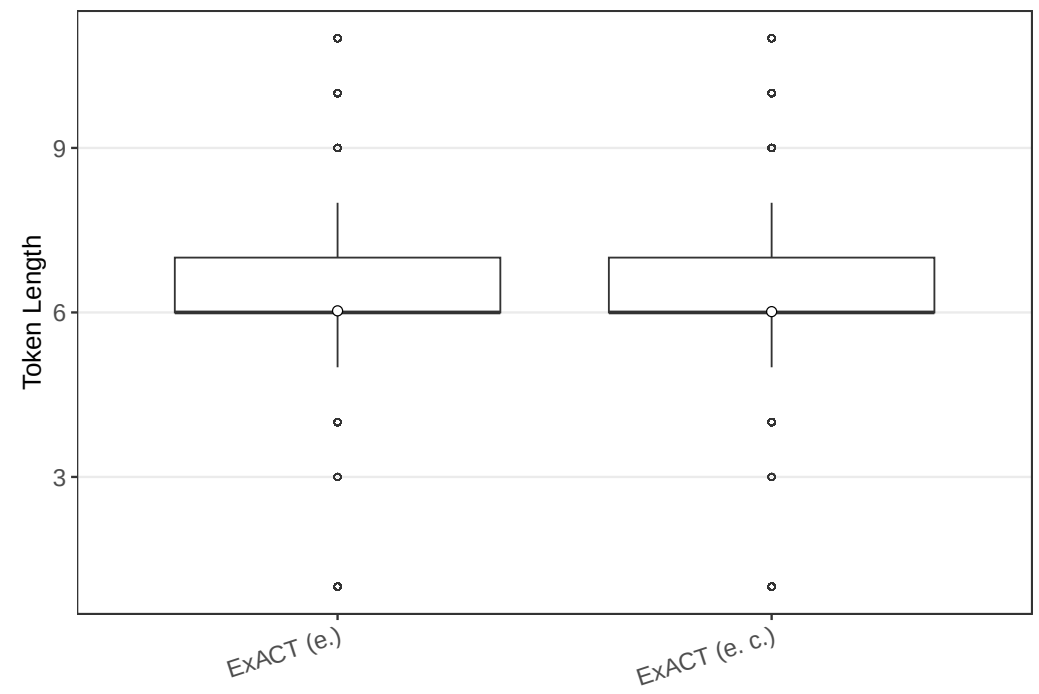

Group	Avg	Median	Min	Max
ExACT (e. c.)	6.01	6	1	11
ExACT (e.)	6.03	6	1	11

Fig. A1 ExACT token length boxplots using explicit and explicit canonical SMILES. The table to the right of the plot shows summary statistics for each representation.

Appendix B Integrating explicitness in atom-in-SMILES

Building on the principles established by ExACT, the integration of explicit chemical information into the AIS method provides a means to encode both atom identity and local connectivity context while retaining the advantages of AIS representation. The atom-in-SMILES-explicit tokenization method extends the conventional AIS method by incorporating all hydrogens, bond orders, branch points, and ring closures from the explicit canonical SMILES. This extension allows each token to preserve the precise local chemical environment of the atom it represents, thus improving the granularity of sequence-based molecular representations without compromising canonical consistency. Additionally, we found that including ring index information should be optional because its relevance varies significantly depending on the dataset. For this reason, in the Results section we present outcomes for both variants: atom-in-SMILES-explicit (AIS-explicit) and atom-in-SMILES-explicit with ring information (AIS-explicit-rings).

The algorithmic procedure for generating AIS-explicit or AIS-explicit-rings tokens is detailed in Algorithm 1. For each atom in the explicit SMILES string, the algorithm accumulates contextual information from preceding bonds, branches, or ring closures and appends it to the token representing the atom. Subsequent atoms inherit relevant contextual markers, ensuring that the sequential representation reflects local molecular topology. Figure B2 illustrates the AIS-explicit and AIS-explicit-rings tokenization

process for a representative molecule, highlighting the propagation of contextual information across adjacent atoms.

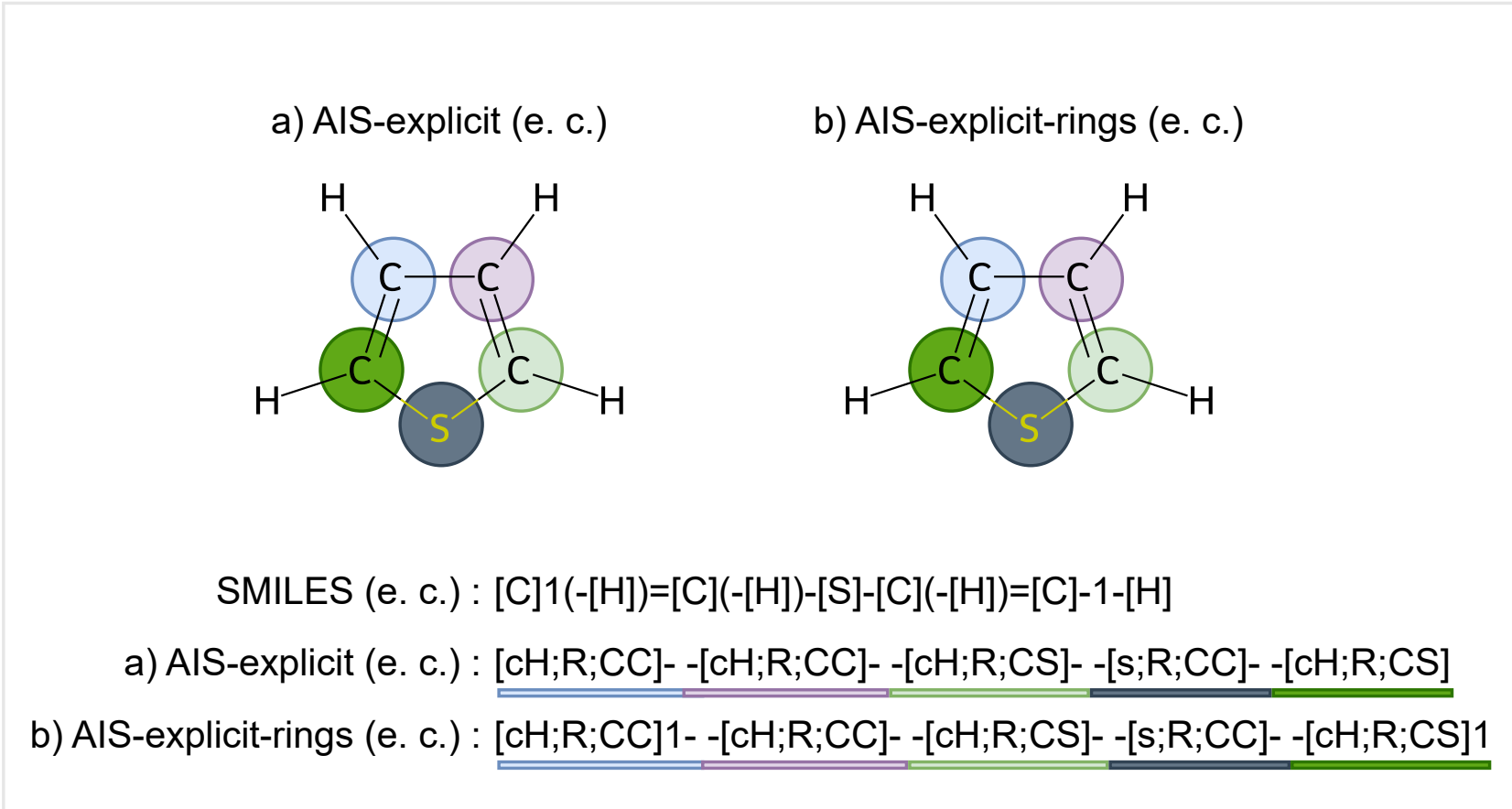

Fig. B2 AIS-explicit and AIS-explicit-rings tokenization example. Parts labeled (a) show AIS-explicit applied to explicit canonical SMILES, while parts labeled (b) show AIS-explicit-rings applied to explicit canonical SMILES.

The observed statistical effects of explicit context integration in AIS tokenization are consistent across both datasets. As shown in Figure B3, the average token length nearly doubles for AIS-explicit and AIS-explicit-rings compared to standard AIS, reflecting the inclusion of additional local connectivity information. The maximum token length increases only slightly, while the minimum token length rises substantially, resulting in a flatter distribution that reduces extreme disparities between short and long tokens. Figure B4 demonstrates that the average token count per molecule is approximately halved for AIS-explicit variants, indicating that context consolidation leads to fewer, but more informative, tokens. Figure B5 illustrates that while AIS-explicit and AIS-explicit-rings exhibit greater variability in unique token counts, the overall distribution is flattened, which promotes a more uniform representation of molecular features across samples. Collectively, these results confirm that explicitness in AIS improves token informativeness and consistency, balancing diversity with representational regularity to facilitate more robust sequence-based modeling.

Appendix C Results for other properties

Algorithm 1 Atom-in-SMILES-explicit algorithm pseudocode

```
Require: Explicit canonical SMILES string S
Ensure: List of tokens Tokens
    parts ← tokenize S into parts using atomInSmiles
    tokens ← []
    current_token ← ""
    current_left_info ← ""
    current_right_info ← ""
    bond_or_branch_detected_flag ← False
    for each part in parts do
        if part is a bond, branch, or ring index symbol then
            bond_or_branch_detected_flag ← True
            current_right_info ← current_right_info + part
        else
            if bond_or_branch_detected_flag then
                current_token ← current_left_info + current_token
                append current_token to tokens
                current_left_info ← current_right_info
                current_right_info ← ""
                current_token ← part
                bond_or_branch_detected_flag ← False
            else
                current_token ← current_token + part
            end if
        end if
    end for
    current_token ← current_left_info + current_token
    append current_token to tokens
    return tokens
```

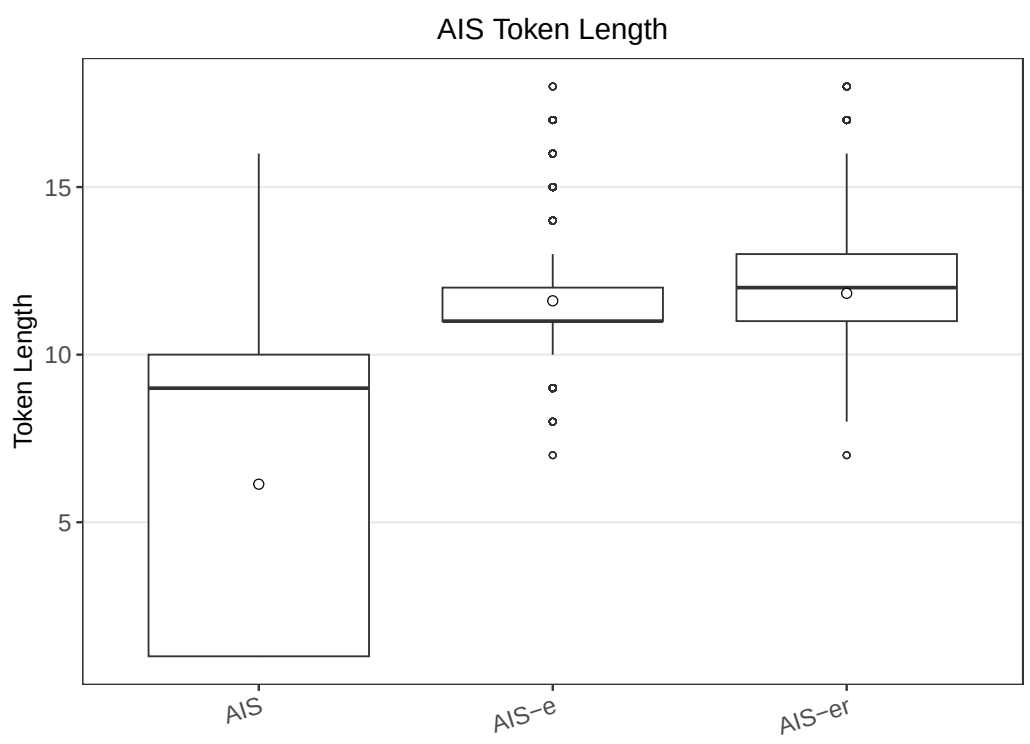

Group	Avg	Median	Min	Max
AIS	6.14	9	1	16
AIS−e	11.61	11	7	18
AIS−er	11.83	12	7	18

Fig. B3 Token length boxplots for AIS, AIS-explicit, and AIS-explicit-rings. The table to the right of the plot shows summary statistics for each tokenization variant.

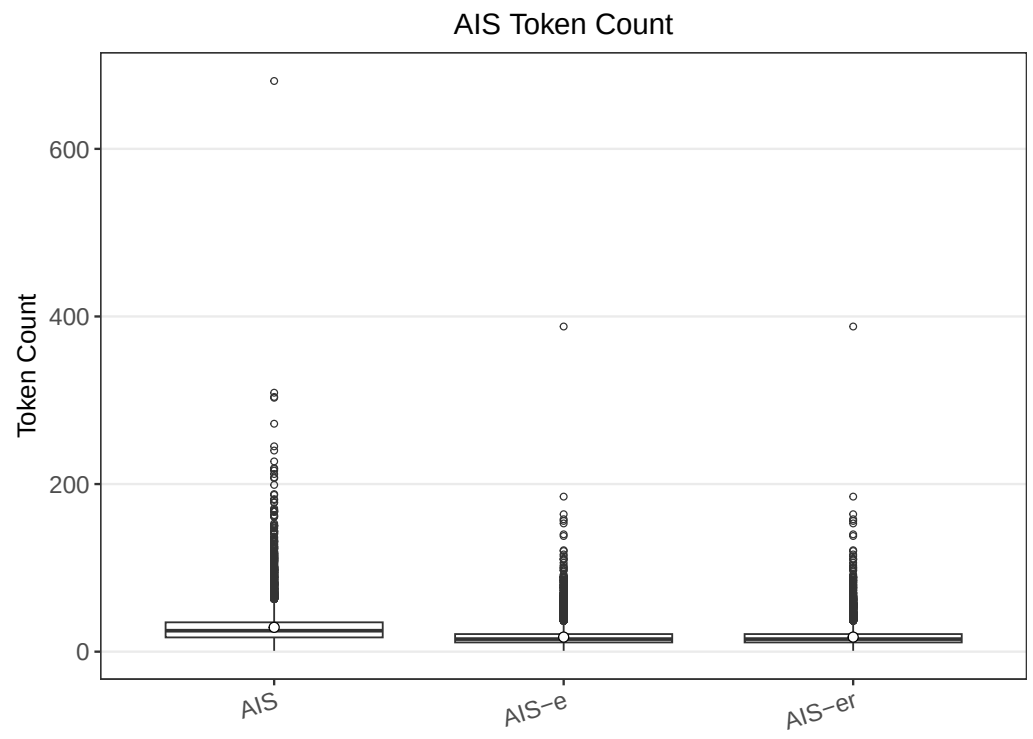

Group	Avg	Median	Min	Max
AIS	29.02	25	1	681
AIS−e	17.38	15	1	388
AIS−er	17.38	15	1	388

Fig. B4 Token count boxplots for AIS, AIS-explicit, and AIS-explicit-rings. The table to the right of the plot shows summary statistics for each tokenization variant.

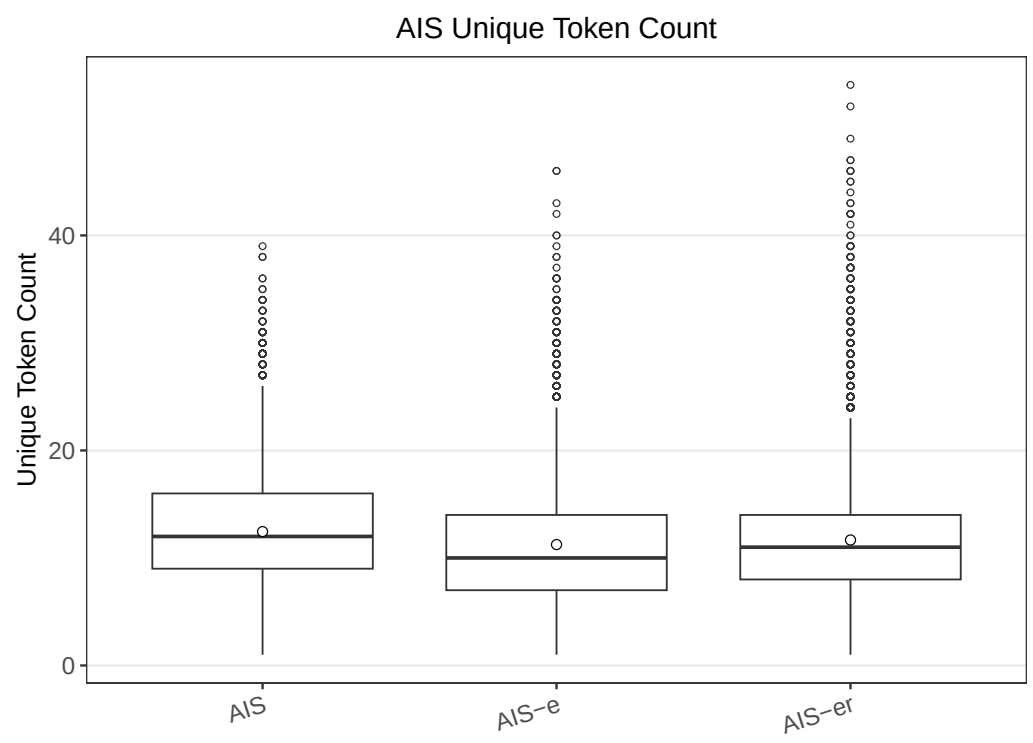

Group	Avg	Median	Min	Max
AIS	12.45	12	1	39
AIS−e	11.25	10	1	46
AIS−er	11.67	11	1	54

Fig. B5 Unique token count boxplots for AIS, AIS-explicit, and AIS-explicit-rings. The table to the right of the plot shows summary statistics for each tokenization variant.

Table C1 Results for adjusted aqueous solubility property. *e.* denotes explicit SMILES, *c.* canonical SMILES and *e. c.* explicit canonical SMILES. AIS-e corresponds to AIS-explicit, and AIS-er to AIS-explicit-rings.

	XGB			CatBoost		
Method	R^2	MAE	$RMSE$	R^2	MAE	$RMSE$
Character	.708 ± .019	.923 ± .022	1.276 ± .039	.725 ± .015	.900 ± .019	1.237 ± .034
Character (c.)	.693 ± .017	.952 ± .021	1.308 ± .035	.709 ± .016	.933 ± .019	1.272 ± .035
Character (e.)	.726 ± .015	.883 ± .019	1.235 ± .034	.739 ± .012	.871 ± .016	1.206 ± .030
Character (e. c.)	.727 ± .015	.881 ± .016	1.233 ± .033	.741 ± .012	.867 ± .016	1.202 ± .029
Atom	.710 ± .017	.927 ± .020	1.271 ± .036	.723 ± .014	.910 ± .016	1.243 ± .031
Atom (c.)	.690 ± .016	.962 ± .017	1.315 ± .031	.702 ± .014	.949 ± .017	1.289 ± .029
Atom (e.)	.722 ± .015	.896 ± .018	1.245 ± .033	.731 ± .013	.889 ± .013	1.225 ± .028
Atom (e. c.)	.724 ± .017	.893 ± .019	1.240 ± .036	.733 ± .014	.887 ± .014	1.220 ± .031
SmilesPE	.591 ± .015	1.148 ± .020	1.510 ± .029	.614 ± .016	1.120 ± .022	1.466 ± .030
SmilesPE (c.)	.567 ± .014	1.190 ± .020	1.554 ± .027	.587 ± .013	1.167 ± .022	1.516 ± .029
SmilesPE (e.)	.720 ± .013	.903 ± .015	1.248 ± .029	.730 ± .013	.894 ± .015	1.226 ± .028
SmilesPE (e. c.)	.724 ± .013	.895 ± .015	1.240 ± .028	.733 ± .013	.888 ± .014	1.220 ± .028
AIS (c.)	**.738 ± .015**	**.864 ± .017**	**1.209 ± .035**	.754 ± .013	.841 ± .016	1.169 ± .031
AIS-e (e. c.)	.730 ± .016	.882 ± .018	1.226 ± .037	.748 ± .012	.856 ± .015	1.184 ± .030
AIS-er (e. c.)	.724 ± .013	.894 ± .017	1.240 ± .030	.741 ± .012	.870 ± .016	1.200 ± .030
ExACT (e.)	.728 ± .018	.890 ± .022	1.230 ± .040	.754 ± .016	.851 ± .018	1.170 ± .036
ExACT (e. c.)	.737 ± .016	.876 ± .020	1.210 ± .036	**.759 ± .014**	**.843 ± .017**	**1.159 ± .031**
MACCs	.726 ± .011	.884 ± .018	1.235 ± .026	.738 ± .011	.873 ± .016	1.209 ± .027
Morgan ($r = 2$)	.662 ± .013	1.006 ± .015	1.373 ± .027	.677 ± .012	.989 ± .014	1.342 ± .027

Table C2 Results for lipophilicity property. *e.* denotes explicit SMILES, *c.* canonical SMILES and *e. c.* explicit canonical SMILES. AIS-e corresponds to AIS-explicit, and AIS-er to AIS-explicit-rings.

	XGB			CatBoost		
Method	R^2	MAE	$RMSE$	R^2	MAE	$RMSE$
Character	.794 ± .043	.773 ± .033	1.593 ± .265	.822 ± .042	.727 ± .034	1.481 ± .262
Character (c.)	.754 ± .045	.845 ± .036	1.742 ± .266	.785 ± .045	.807 ± .034	1.629 ± .270
Character (e.)	.780 ± .048	.768 ± .033	1.646 ± .276	.812 ± .048	.737 ± .033	1.522 ± .286
Character (e. c.)	.786 ± .046	.763 ± .032	1.625 ± .276	.809 ± .046	.743 ± .033	1.532 ± .282
Atom	.791 ± .047	.771 ± .034	1.606 ± .278	.817 ± .040	.729 ± .031	1.502 ± .254
Atom (c.)	.749 ± .047	.850 ± .034	1.757 ± .269	.779 ± .043	.812 ± .033	1.648 ± .261
Atom (e.)	.761 ± .052	.802 ± .037	1.714 ± .285	.791 ± .047	.776 ± .031	1.604 ± .277
Atom (e. c.)	.763 ± .053	.800 ± .034	1.709 ± .289	.791 ± .048	.780 ± .032	1.604 ± .282
SmilesPE	.717 ± .057	1.053 ± .037	1.867 ± .301	.744 ± .054	1.018 ± .036	1.776 ± .289
SmilesPE (c.)	.680 ± .071	1.111 ± .038	1.983 ± .337	.715 ± .055	1.080 ± .039	1.874 ± .288
SmilesPE (e.)	.766 ± .053	.797 ± .032	1.698 ± .291	.798 ± .047	.769 ± .031	1.578 ± .277
SmilesPE (e. c.)	.764 ± .060	.793 ± .034	1.701 ± .312	.797 ± .049	.769 ± .028	1.582 ± .283
AIS (c.)	.767 ± .058	.754 ± .032	1.692 ± .316	.801 ± .050	.705 ± .030	1.563 ± .292
AIS-e (e. c.)	**.823 ± .058**	**.651 ± .035**	**1.474 ± .330**	.853 ± .050	.606 ± .031	1.342 ± .308
AIS-er (e. c.)	.814 ± .059	.678 ± .037	1.508 ± .332	.849 ± .049	.632 ± .032	1.360 ± .301
ExACT (e.)	.799 ± .052	.702 ± .035	1.573 ± .299	.848 ± .042	.606 ± .027	1.365 ± .270
ExACT (e. c.)	.809 ± .053	.683 ± .036	1.533 ± .305	**.853 ± .047**	**.595 ± .028**	**1.340 ± .297**
MACCs	.726 ± .034	.902 ± .031	1.838 ± .203	.740 ± .036	.873 ± .031	1.789 ± .220
Morgan ($r = 2$)	.711 ± .040	.979 ± .035	1.887 ± .240	.716 ± .045	.984 ± .029	1.871 ± .258

Table C3 Results for molar refractivity property. *e.* denotes explicit SMILES, *c.* canonical SMILES and *e. c.* explicit canonical SMILES. AIS-e corresponds to AIS-explicit, and AIS-er to AIS-explicit-rings.

	XGB			CatBoost		
Method	R^2	MAE	$RMSE$	R^2	MAE	$RMSE$
Character	$.824 \pm .074$	$7.59 \pm .42$	19.25 ± 5.14	$.837 \pm .072$	$7.12 \pm .35$	18.59 ± 5.82
Character (c.)	$.671 \pm .084$	$11.18 \pm .52$	26.61 ± 6.07	$.733 \pm .080$	$10.68 \pm .40$	23.97 ± 5.97
Character (e.)	$.722 \pm .082$	$8.79 \pm .48$	24.44 ± 6.01	$.765 \pm .089$	$8.59 \pm .42$	22.43 ± 6.46
Character (e. c.)	$.723 \pm .086$	$8.89 \pm .51$	24.36 ± 6.17	$.763 \pm .088$	$8.75 \pm .37$	22.55 ± 6.35
Atom	$.823 \pm .056$	$7.41 \pm .31$	19.48 ± 4.83	$.830 \pm .080$	$7.14 \pm .35$	18.97 ± 6.24
Atom (c.)	$.673 \pm .074$	$10.91 \pm .42$	26.57 ± 5.67	$.723 \pm .080$	$10.85 \pm .41$	24.42 ± 5.97
Atom (e.)	$.711 \pm .097$	$9.31 \pm .53$	24.91 ± 6.64	$.746 \pm .092$	$9.39 \pm .48$	23.35 ± 6.53
Atom (e. c.)	$.706 \pm .091$	$9.52 \pm .47$	25.16 ± 6.42	$.746 \pm .092$	$9.60 \pm .48$	23.32 ± 6.53
SmilesPE	$.721 \pm .110$	$12.26 \pm .42$	24.44 ± 7.14	$.750 \pm .108$	$12.11 \pm .45$	23.08 ± 7.15
SmilesPE (c.)	$.555 \pm .116$	$14.88 \pm .51$	30.81 ± 6.47	$.619 \pm .102$	$14.57 \pm .45$	28.53 ± 6.18
SmilesPE (e.)	$.703 \pm .091$	$9.54 \pm .51$	25.27 ± 6.34	$.755 \pm .091$	$9.49 \pm .43$	22.90 ± 6.48
SmilesPE (e. c.)	$.705 \pm .087$	$9.72 \pm .48$	25.19 ± 6.18	$.750 \pm .092$	$9.62 \pm .42$	23.14 ± 6.53
AIS (c.)	$.688 \pm .086$	$10.01 \pm .38$	25.91 ± 6.16	$.758 \pm .095$	$9.52 \pm .40$	22.77 ± 6.72
AIS-e (e. c.)	$.776 \pm .099$	$8.16 \pm .58$	21.87 ± 7.13	$.830 \pm .091$	$7.11 \pm .50$	18.93 ± 7.00
AIS-er (e. c.)	$.781 \pm .100$	$8.12 \pm .53$	21.60 ± 7.12	$.827 \pm .094$	$7.30 \pm .44$	19.14 ± 7.06
ExACT (e.)	$.776 \pm .084$	$\mathbf{6.34 \pm .48}$	21.92 ± 6.36	$\mathbf{.851 \pm .097}$	$\mathbf{4.96 \pm .34}$	$\mathbf{17.60 \pm 7.37}$
ExACT (e. c.)	$\mathbf{.782 \pm .097}$	$6.24 \pm .54$	$\mathbf{21.56 \pm 7.00}$	$.849 \pm .097$	$5.01 \pm .35$	17.69 ± 7.40
MACCs	$.689 \pm .083$	$11.10 \pm .52$	25.77 ± 5.24	$.721 \pm .074$	$10.74 \pm .42$	24.50 ± 5.66
Morgan ($r = 2$)	$.631 \pm .074$	$13.16 \pm .52$	28.18 ± 5.62	$.658 \pm .073$	$13.11 \pm .45$	27.18 ± 5.67

Appendix D Chemical diversity of AqSolDB

As shown in Figure D6, the filtered AqSolDB dataset covers a wide range of structural and physicochemical characteristics, with considerable variation in molecular topology, hydrogen-bonding properties, polarity, lipophilicity, and molar refractivity. This diversity suggests that the dataset encompasses chemically heterogeneous compounds suitable for evaluating molecular representation methods.

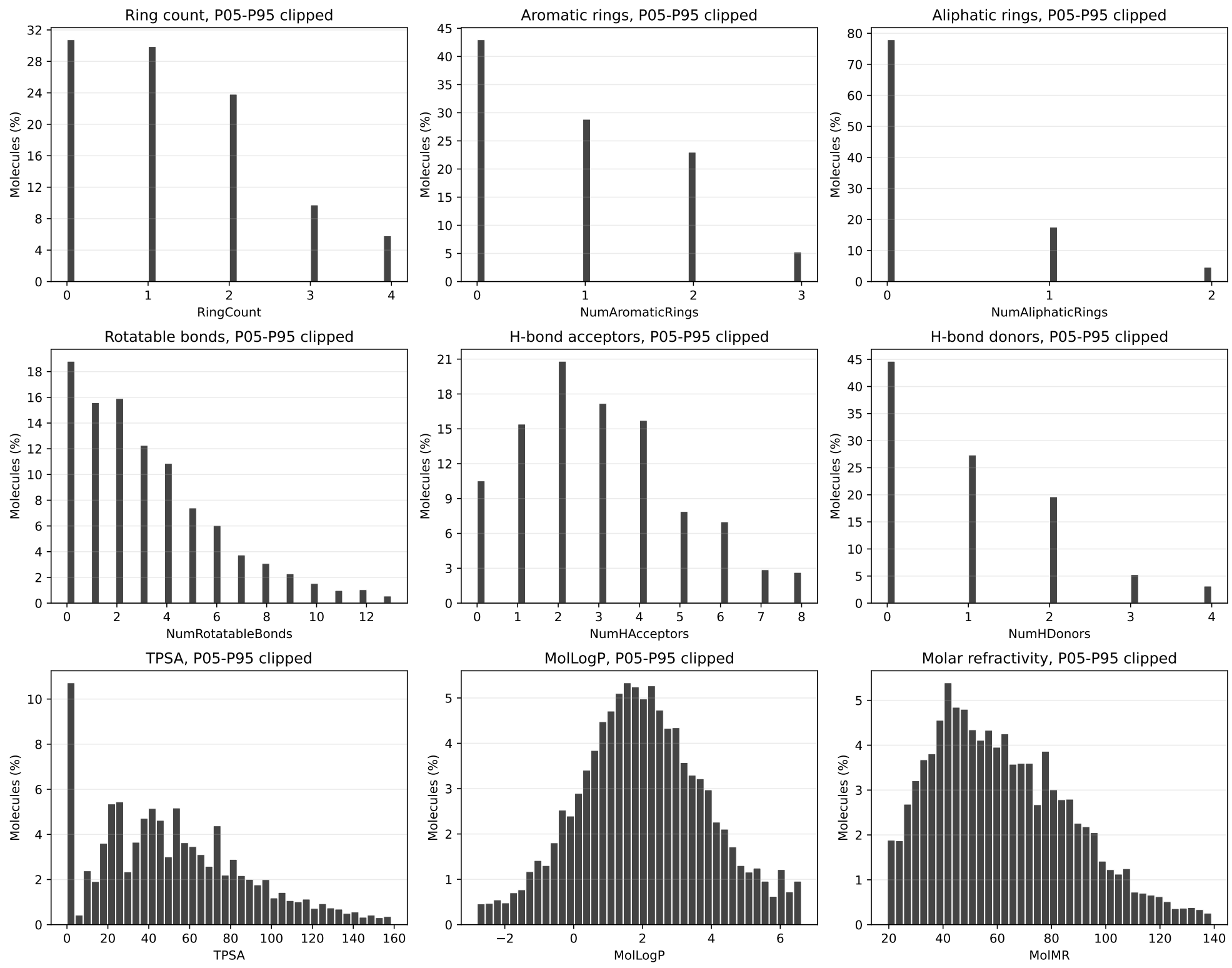

Fig. D6 Structural and physicochemical characteristics distributions for the filtered AqSolDB dataset. Histograms show the percentage of molecules in each descriptor bin after clipping values to the 5th–95th percentile range to reduce the visual influence of extreme outliers.
